# Cigarette and Waterpipe Smoking Decrease Respiratory Quality of Life in Adults: Results from a National Cross-Sectional Study

**DOI:** 10.1155/2012/868294

**Published:** 2012-09-03

**Authors:** Salamé Joseph, Salameh Pascale, Khayat Georges, Waked Mirna

**Affiliations:** ^1^Faculty of Medical Sciences, Lebanese University, Beirut 961, Lebanon; ^2^Faculties of Pharmacy and of Public Health, Lebanese University, Beirut 961, Lebanon; ^3^Hôtel-Dieu de France Hospital and Faculty of Medicine, Saint Joseph University, Beirut 961, Lebanon; ^4^Saint George Hospital University Medical Center and Faculty of Medicine, Univerity of Balamand, Beirut 961, Lebanon

## Abstract

*Background*. Chronic obstructive pulmonary disease (COPD) is gaining an importance over the world, and its effect on quality of life is better grasped. Our objective was to use the Clinical COPD Questionnaire (CCQ) to describe the respiratory quality of life in the Lebanese population, stressing on differences between smokers and nonsmokers. *Methods*. Using data from a cross-sectional national study, we checked the construct validity and reliability of the CCQ. Factors and items correlation with postbronchodilator FEV1/FVC were reported, in addition to factors and scale association with COPD and its severity. We then conducted a multiple regression to find predictors of quality of life. *Results*. The CCQ demonstrated excellent psychometric properties, with adequacy to the sample and high consistency. Smokers had a decreased respiratory quality of life versus nonsmokers, independently of their respiratory disease status and severity. This finding was confirmed in COPD individuals, where several environmental factors, lower education, and cumulative smoking of cigarette and of waterpipe were found to be independent predictors of a lower quality of life, after adjusting for COPD severity. *Conclusions*. Smoking decreases the respiratory quality of life of Lebanese adults; this issue has to be further emphasized during smoking cessation and patients' education.

## 1. Introduction

Chronic obstructive pulmonary disease is increasing over the world; it is expected to rank third in 2020 as a cause of mortality [1]. In Lebanon, we had demonstrated that the prevalence of respiratory diseases is quite high (COPD and chronic bronchitis in particular) in the population aged 40 and above, paralleling the high prevalence of smoking cigarettes and waterpipes [2]. Although COPD is known to decrease the patients' quality of life [3], a low percentage (20%) of individuals are diagnosed and treated for COPD in Lebanon. The others are still experiencing chronically respiratory symptoms and consequent limitations without seeking help [2]. However, according to the GOLD (Global initiative for obstructive lung disease) guidelines [4] and their updated version [5], the aim of clinical control in patients with COPD includes health-related quality of life goals (improved exercise tolerance and emotional function) added to clinical goals (prevention of disease progression and minimization of symptoms).

Several tools have been developed to evaluate quality of life in patients with chronic respiratory diseases [6, 7], but none of them has been validated for use in the Lebanese population. Moreover, the Saint George Respiratory Questionnaire is long [8], while the American Thoracic Society questionnaire evaluates only symptoms [9]. However, the Chronic COPD Questionnaire (CCQ) seems to have excellent psychometric properties, along with simplicity of application [10]; it is also the first questionnaire that incorporates both clinician and patient guideline goals in the clinical control evaluation of patients with COPD in general clinical practice [11]. It was showed to be the best patient-reported outcome tool to assess functional performance [12]. Although the new recommendations issued by the GOLD steering committee [5] adopted the COPD Assessment Test (CAT) questionnaire as the first one to be used without neglecting the value of the CCQ, we had conducted the study before the issuing of these recommendations.

Our objective was to describe the respiratory quality of life in the Lebanese population using data from a cross-sectional national study on the prevalence of COPD [2], stressing on differences between smokers and nonsmokers. 

## 2. Methods

### 2.1. Study Design

Data for this analysis was taken from a cross-sectional study, using a multistage cluster sample all over Lebanon. This study was carried out between October 2009 and September 2010, using a multistage cluster sample (*n* = 2201) across Lebanon. From the list of communities in Lebanon (includes a total of 2782 villages, towns, and cities), one hundred communities were randomly selected with randomization performed on a computerized software. Afterwards, through a representative of local authorities, individuals were randomly chosen to be interviewed, from a provided list of dwelling households aged 40 years and above. All individuals of the household were solicited, if they were eligible. 

### 2.2. Procedure

After an oral informed consent, subjects underwent a baseline spirometry (Micro Lab, Micro Medical Limited, UK), conducted by a trained technician, and answered a standardized questionnaire. Thirty minutes after the inhalation of 2 puffs of ipratropium bromide (18 *μ*g/actuation) and albuterol sulfate (103 *μ*g/actuation) (Combivent) in a pressurised metered-dose aerosol unit, a postbronchodilator spirometry was performed. The best result out of 3 trials was taken into account. Spirometric quality was checked, and FEV6/FVC was ≤100% in more than 99.2% of measurements. Additional methodological details are presented in a separate publication [2]. 

### 2.3. Questionnaire and Procedure

The standardized questionnaire included sections about sociodemographic characteristics, respiratory diseases and symptoms, and a thorough smoking history evaluation. Moreover, respiratory-related quality of life was measured by the Clinical COPD Questionnaire (CCQ) [10], while the MRC dyspnea scale was used to evaluate dyspnea [13]. The questionnaires were administered in Arabic local language; the translation process was as follows: first, two of the researchers, both bilingual, forward translated the questions into Arabic; instructions were given to them in the approach to translating, emphasizing conceptual rather than literal translations, as well as the need to use natural and acceptable language for the broadest audience. Second, discrepancies were resolved by consensus between them and two other researchers: this panel thus included the original translators, experts in health, as well as experts with experience in instrument development and translation. Third, an independent translator with no knowledge of the questionnaire back translated the questions into English. Translation discrepancies were resolved by consensus between the researchers and the translator. Fourth, the questionnaire was pilot tested on 20 individuals; all questions were deemed clear by these individuals, and no further changes were made to the initial questions. 

### 2.4. Definitions

Chronic Obstructive Pulmonary Disease (COPD) was defined and classified according to GOLD guidelines (FEV1/FVC < 0.70 postbronchodilator) [14], and according to the lower limit of normal (FEV1/FVC postbronchodilator < 5th percentile of the healthy population having the same age and gender of the individual) [15]. Individuals were finally classified as having COPD if they fulfilled one of the definitions described above. Chronic bronchitis was defined by the declaration of morning cough and expectorations for more than 3 months a year over more than two years in individuals with no COPD [14]. On the other hand, an individual was considered “healthy” from the respiratory point of view if he had no respiratory symptoms and no respiratory disease. Moreover, patients with a partially reversible obstruction (postbronchodilator FEV1/FVC that does not go back to normal) are considered with a mixed disorder of asthma and COPD; they are termed “reversible COPD.” Further methodology details are presented in another publication [2]. 

Cumulative dosing of cigarettes was calculated as the mean number of daily packs multiplied by the duration of smoking (pack∗years), while that of waterpipe was calculated as the mean number of weekly waterpipes multiplied by the duration of smoking (waterpipe∗years). Cigarette and waterpipe dependence were defined according to Fagerström Test for Nicotine Dependence (FTND) [16] and Lebanese Waterpipe Dependence Scale (LWDS-11) [17], respectively. 

### 2.5. Statistical Analysis

SPSS version 17.0 was used to enter and analyze data. Weighting was performed according to the numbers published by the Lebanese Central Administration of Statistics in 2007, taking into account gender, age, and dwelling region [18]. Cluster effect was taken into account, according to Rumeau-Rouquette and collaborators [19]. 

A *P* value of 0.05 was considered significant. The Chi^2^ test was used for cross tabulation of qualitative variables in bivariate analysis, and odds ratios (OR) were calculated. ANOVA and Kruskal-Wallis tests were used to compare between three groups or more, and Pearson correlation coefficient were used to correlate between quantitative variables. Bonferroni adjustment was used for ANOVA post hoc tests of between groups comparison. 

To confirm the CCQ construct validity in the Lebanese population, a factorial analysis was launched for CCQ items, using the principal component analysis technique, with a promax rotation since the extracted factors were found to be significantly correlated. The Kaiser-Meyer-Olkin measure of sampling adequacy and Bartlett's test of sphericity were ensured to be adequate. The retained number of factors corresponded to Eigenvalues higher than one. Factors loading of items were recorded. Moreover, Cronbach's alphas were recorded for reliability analysis for the total score and for subscale factors. The total CCQ score represents the sum of the 10 CCQ items divided by 10 (as recommended in the CCQ manual) [10], while the factors 1 & 2 are the sums of their respective items. Factors and items correlation with postbronchodilator spirometric FEV1/FVC were reported, in addition to factors and scale association with COPD and its severity. 

Afterwards, backward linear multiple regression was performed for multivariate analyses, with CCQ score as the dependent variable, and sociodemographic characteristics and other potentially harmful exposures as the independent variables; after ensuring model adequacy to data, relationship linearity, dependent variable normality, and lack of collinearity between covariates. We used this method to find significant predictors of respiratory quality of life in all individuals, in patients with COPD and in nonsmokers. Moreover, partial correlation with CCQ score was presented, taking other covariates into account. 

## 3. Results

### 3.1. Sample Description

Among 2201 individuals, 978 were considered healthy (44.5%) from the respiratory point of view. Moreover, 233 (10.6%) had COPD, 204 (9.3%) had asthma, 51 (2.3%) had a reversible COPD, 326 (14.8%) had chronic bronchitis, 72 (3.3%) had a restrictive disease, and 336 individuals (15.3%) had miscellaneous respiratory symptoms (MRS). In the following analysis we will exclude patients with asthma, restrictive disease, and miscellaneous respiratory symptoms. 

Thus, patients with COPD (*n* = 284), chronic bronchitis (*n* = 326), and healthy individuals (*n* = 978) will only be included. For them, mean postbronchodilators FEV1/FVC significantly differed: 0.62 (SD = 0.09) for COPD patients, 0.83 (SD = 0.05) for chronic bronchitis, and 0.85 (SD = 0.03) for healthy individuals (*P* < 0.001 for all comparisons). Healthy individuals have never been hospitalized for respiratory problems, while COPD and chronic bronchitis patients have both been hospitalized (mean number of hospitalizations is 0.26 for COPD and 0.36 for chronic bronchitis; *P* = 0.090). 

### 3.2. Sociodemographic and Health Characteristics

In Table 1, we present sociodemographic characteristics of different individuals' categories. We note significant differences in percentages of COPD and chronic bronchitis for all categories. Individuals with obstructive diseases were included older ages, more males, less educated, retired, nonmarried, more obese individuals with more cardiac problems, in the regions of Bekaa and South Lebanon (*P* < 0.001 for all). Moreover, 22.2% of patients with COPD and 17.2% of those with chronic bronchitis are getting inhalation therapy.

Smokers had the higher rates of COPD and of chronic bronchitis, compared with never smokers. While mixed smokers had significantly higher prevalences of both diseases versus exclusive smokers, current waterpipe smokers had rates similar to never smokers, while previous waterpipe smokers included more COPD than previous cigarette smokers, with no chronic bronchitis cases (Table 1). 

### 3.3. Clinical COPD Questionnaire (CCQ) Factor Analyses

Although the CCQ questions were part of the cross-sectional study questionnaire, and they were asked to the whole sample, the factorial analysis that was run over the sample of healthy individuals, COPD and chronic bronchitis patients (Total *n* = 1588). CCQ items converged over a solution of two factors that had an Eigenvalue over 1, explaining a total of 67.91% of the variance. A Kaiser-Meyer-Olkin measure of sampling adequacy of 0.876 was found, with a significant Bartlett's test of sphericity (*P* < 0.0001). 

The first one, representing “dyspnea and dysfunction”, explained 56.30% of the variance; the second factor, representing “chronic bronchitis” explained 11.61% of the variance. Moreover, high Cronbach's alpha were found for factor 1 (0.909), factor 2 (0.859), and the full scale (0.910) (Table 2). 

### 3.4. Quality of Life in Disease Categories

There were significant differences between the means of respiratory quality of life score (Table 3) (*P* < 0.001). Looking at the means, the lowest CCQ quality of life was found for reversible and irreversible COPD patients and chronic bronchitis, compared with healthy individuals. We also compared respiratory CCQ score in COPD grades: there was a significant increase in CCQ along with COPD severity grades (*P* < 0.001). In individuals declaring being treated by inhalation therapy (including short acting and long acting anticholinergics, beta agonists, and steroids), quality of life was significantly lower versus individuals not declaring so (*P* < 0.001). Moreover, we found a significant correlation between the CCQ and the MRC dyspnea scale (*r* = 0.763; *P* < 0.001); individuals with an MRC dyspnea scale higher than zero had significantly worse quality of life (Table 3). 

### 3.5. Quality of Life and Smoking

For previous smoking, we note significantly a higher CCQ score for all types of smoking, including cigarette, waterpipe, and mixed smoking (*P* < 0.001), compared with never smokers; mixed smokers have significantly higher CCQ versus other categories, while cigarette and waterpipe smoking had nonsignificant differences. As for current smoking, no significant difference was found between waterpipe smoking and never smokers; however, cigarette and mixed smokers had significantly higher sores for CCQ (Table 4). 

For patients with chronic bronchitis and COPD, any previous smoker had significantly lower CCQ versus never smokers; mixed smokers had significantly higher values than cigarette and never smokers. In current smokers, cigarette and mixed smokers had significantly higher QOL versus waterpipe and never smokers. No significant difference was found between never smokers and waterpipe smokers, and no significant difference was found between cigarette and mixed smokers (Table 4). 

On the other hand, there were clear positive dose-effect relationships between different smoking types cumulative doses and quality of life score (the higher the cumulative dose of smoking, the lower the quality of life): correlation coefficients between CCQ and cumulative doses were all positive (*P* < 0.001); CCQ means differed for previous and current cigarette smoking, for previous and current waterpipe smoking, and for current cigarette and waterpipe dependence classes (*P* < 0.001). Again, similar results are found for COPD and chronic bronchitis patients (Table 5). 

### 3.6. Predictors of Quality of Life

Predictors of respiratory quality of life, measured by CCQ, are presented in Table 6, by decreasing order of importance: cumulative cigarette dose, older age, having at least one smoker in the family, lower education, female gender, any heart disease, heating house by diesel, cumulative waterpipe dose, heating house by hot air, and having at least one smoker at work were significant predictors of a lower respiratory quality of life (higher CCQ score; *P* < 0.05 for all); ever living close to a local power plant (electricity generator) was important but its effect did not reach statistical significance (*P* < 0.10) (Table 6). 

In COPD individuals, by decreasing order of importance, CCQ was significantly affected by cumulative cigarette dose, declared inhalation therapy, female gender, lower education, having at least one smoker in the family, older age, cumulative waterpipe dose, having a cardiac problem, not heating home centrally, and COPD severity grading (Table 6). 

Finally, we present in a multivariate analysis the predictors of quality of life in never smokers, by decreasing order of importance. We found that lower education, having a cardiac problem, heating home by hot air, older age, heating its house by diesel, ever living close to a heavy traffic road (<100 m), and occupational exposure to toxic fumes were all significantly associated with a lower quality of life; having at least one smoker in the family was important but their effect did not reach statistical significance (*P* ≤ 0.10) (Table 6). 

In the study sample (healthy, COPD and chronic bronchitis individuals), cumulative dosing of cigarettes (*r* = 0.404; *P* < 0.001) and cumulative dosing of waterpipe (*r* = 0.078; *P* < 0.001) were both significantly correlated with CCQ score. In the COPD and chronic bronchitis subgroup, these values were, respectively:  *r* = 0.263 (*P* < 0.001) and *r* = 0.103 (*P* = 0.003). 

## 4. Discussion

In this study, we were able to describe the quality of life of Lebanese residents aged 40 years and more. The CCQ demonstrated excellent psychometric properties, with an excellent adequacy to a cross-sectional sample and high consistency. As expected, the respiratory related quality of life of COPD patients was decreased relative to healthy individuals; in addition, patients with chronic bronchitis without COPD and reversible COPD disorders also demonstrated a lower quality of life versus healthy individuals. These results have already been found by others: Weatherall and collaborators' work for COPD [20], and Maleki-Yazdi and collaborators' [21] for chronic bronchitis are some examples. 

There was also significantly lower quality of life in previous and current smokers in the same disease category versus nonsmokers; one exception is for current smokers of waterpipe. This could be explained with the fact that waterpipe smoking in Lebanon is a relatively new trend, with the majority of waterpipe smokers having a low duration of smoking. However, a dose-effect relationship was clear for the effect of all types of smoking on QOL, with lower quality of life scores in patients with heavier smoking cumulative doses; this result was even found for current waterpipe smokers. Smokers had a decreased respiratory quality of life versus nonsmokers, independently of their respiratory disease. The association of cigarette smoking with lower quality of life has been found by Kotz and collaborators using the CCQ [12], and by Geijer and collaborators, where smoking induced limitations of physical functioning [22]; it was also indirectly shown by Papadopoulos and collaborators, with smoking cessation improving quality of life [23]. For waterpipe, this association seems of lower magnitude; nevertheless, it has been demonstrated by Tavafian and collaborators using the SF-36 [24]. 

The relationship between other factors and lower quality of life was also confirmed in COPD individuals: besides cumulative smoking of cigarette and of waterpipe that was previously discussed, several indoor and outdoor environmental factors, age, gender, and lower education were found to be independent predictors of a lower quality of life, after adjusting for COPD severity grades. In fact, it has been shown that persons who have similar reductions in forced expiratory volume in 1st second and exercise capacity and similar levels of dyspnea have a wide range of HRQL, suggesting that other variables contributed to quality of life, such as age and gender [25]. 

In never smokers, older age, lower education, having a cardiac problem, heating its house by hot air or by diesel, occupational exposure to toxic fumes, ever living close to a heavy traffic road, and having at least one smoker in the family were all associated with a lower respiratory quality of life. We had already showed that these factors were independently associated with chronic bronchitis [26] and COPD [2]; this may explain their association with lower respiratory quality of life. 

One noticeable result is the lower quality of life in individuals declaring being treated with inhalation therapy; one explanation could be the fact that patients who are more symptomatic in general are the ones who go and seek a physician's help. In fact, in our study, patients with COPD and chronic bronchitis who admitted being treated by inhaled therapy also declared having more chronic cough, expectorations, and wheezing than those without therapy; they also had more severe disease staging (results not shown). This issue may further be explained by the delay in diagnosis and treatment of individuals, the noncompliance to treatment of some individuals, and the irreversible nature of the disease. Additional studies are necessary to clarify this point. 

Despite excellent results in this epidemiological setting, the value of the CCQ scale to evaluate the respiratory quality of life in the Lebanese population should additionally be tested in clinical settings. Moreover, we suggest a comparison of performance with the CAT scale that was shown to be superior to CCQ as a tool for monitoring the impact of symptom variability on the lives of patients with COPD [5]. Other limitations of our work include a possibility of selection bias, and information bias coupled with the used questionnaire. However, the demonstrated dose-effect relationship and the multivariate analyses are considered strong points of this work. Nevertheless, given this data was collected from a Lebanese population, predictive factors native to the Mediterranean region such as smoking a waterpipe may not be generalized to the general worldwide population.

## 5. Conclusions

In conclusion, we were able to describe the respiratory quality of life of Lebanese residents aged 40 years or more, using a valid tool. We found a lower quality of life in smokers versus nonsmokers, even in the same respiratory disease category and severity grade. A dose-effect relationship was also shown with lower quality of life with higher severity of the disease and higher cumulative smoking. This issue should be further emphasized during patients' education and smoking cessation. 

## Figures and Tables

**Table 1:** Sociodemographic characteristics of the study population.

Characteristic	Healthy (*n* = 978)	COPD (reversible and irreversible) (*n* = 284)	Chronic bronchitis without COPD (*n* = 326)	Total* (*n* = 1588)
Region				
Beirut	57.4%	21.3%	21.3%	277
Mount Lebanon	67.2%	14.8%	17.9%	687
North Lebanon	67.3%	17.1%	15.6%	263
South Lebanon	55.6%	15.3%	29.1%	196
Bekaa plain	43.0%	29.1%	27.9%	165
Gender				
Male	58.9%	20.9%	20.2%	774
Female	64.2%	14.9%	20.9%	812
Age class				
40–44 years	79.4%	6.2%	14.3%	321
45–49 years	78.6%	9.0%	12.4%	266
50–54 years	70.0%	11.5%	18.5%	227
55–59 years	58.8%	16.1%	25.1%	199
60–64 years	48.2%	29.9%	21.8%	197
65 years and more	37.8%	32.5%	29.6%	378
Education				
Illiterate	50.5%	17.6%	31.9%	91
<8 years of school	46.9%	22.1%	31.0%	290
8−12 years of school	54.2%	24.5%	21.4%	323
12.1–15 years of school	61.3%	21.3%	17.4%	432
University studies	79.1%	7.3%	13.6%	441
Work status				
Currently working	71.5%	13.1%	15.4%	846
Retired	42.0%	31.2%	26.8%	231
Not finding a job	66.7%	20.0%	13.3%	15
Do never work	53.6%	19.8%	26.6%	496
Marital status				
Married	62.2%	17.5%	20.3%	1303
Single	64.1%	12.8%	23.1%	156
Widow or divorced	51.2%	28.1%	20.7%	121
Body Mass Index				
No obesity	62.7%	17.8%	19.5%	1261
Obesity	53.8%	20.2%	25.9%	247
Cardiac problem				
No	66.1%	16.2%	17.7%	1331
Yes	38.4%	26.4%	35.3%	258
Inhalation therapy				
No	66.6%	15.0%	18.4%	1477
Yes	0	52.9%	47.1%	111
Current smoking				
Never smokers	84.1%	4.5%	11.4%	552
Cigarette smokers	45.3%	24.6%	30.1%	479
Waterpipe smokers	83.7%	4.8%	11.5%	104
Mixed smokers	50.6%	27.2%	22.2%	81
Previous smokers				
Never smokers	84.1%	4.5%	11.4%	552
Cigarette smokers	46.8%	26.9%	26.3%	308
Waterpipe smokers	65.5%	34.5%	0	55
Mixed smokers	30.0%	42.9%	27.1%	70

*All *P* values were <0.001.

**Table 2:** Factorial analysis of the Clinical COPD Questionnaire.

Items	Factor loading	Factors correlation*	Correlation with FEV1/FVC*
Factor 1**		Factor 1	−0.436
Had dyspnea at rest	0.480	0.771	−0.356
Had dyspnea on effort	0.607	0.876	−0.422
Was unable to do strenuous effort such as going up stairs	0.701	0.871	−0.451
Was unable to do moderate effort such as walking	0.776	0.896	−0.430
Was anxious about breathing difficulties or getting a cold	0.671	0.786	−0.340
Was depressed because of respiratory problems	0.715	0.733	−0.227
Was unable to socialize (talking, visiting,…)	0.949	0.767	−0.291
Was unable to do daily activities/dressing …	0.925	0.819	−0.313

Factor 2**		Factor 2	−0.442
Had sputum production	0.980	0.959	−0.414
Had cough	0.937	0.956	−0.439

Total scale			−0.464

*All correlations were significant (*P* < 0.001); factor 1 correlation with CCQ was 0.980; factor 2 correlation with CCQ was 0.829; **Cronbach's alpha = 0.910 for the full scale, 0.909 for factor 1 and 0.859 for factor 2; factor 1 correlation coefficient with factor 2 was 0.700.

**Table 3:** Respiratory-related quality of life (CCQ^1^) scores.

Categories	Number	Score mean	Score standard deviation
Respiratory diseases			
Healthy	978	0.31	0.60
COPD^2^	233	2.45	1.50
Chronic bronchitis	326	2.12	1.61
Reversible COPD^2^	51	2.06	1.76
Total	1588	1.05	1.44
*P* value for ANOVA^3^	<0.001		
COPD grades^4^			
Grade 1 (FEV1 ≥ 0.8)	37	2.48	1.39
Grade 2 (0.5 ≤ FEV1 < 0.8)	124	2.44	1.49
Grade 3 (0.3 ≤ FEV1 < 0.5)	43	3.03	1.55
Grade 4 (FEV1 < 0.3)	8	3.63	1.68
*P* value for ANOVA	<0.001		
Individuals with all COPD^5^			
Taking inhalation therapy	63	3.00	1.48
Not taking inhalation therapy	221	2.21	1.53
*P* value for ANOVA	<0.001		
Individuals with chronic bronchitis			
Taking inhalation therapy	56	3.42	1.60
Not taking inhalation therapy	270	1.85	1.48
*P* value for ANOVA	<0.001		
MRC dyspnea scale^6^			
MRC = 0	999	0.33	0.67
MRC > 0	589	2.27	1.57
*P* value for ANOVA	<0.001		

^1^CCQ: Clinical COPD Questionnaire; ^2 ^COPD: Chronic Obstructive Pulmonary Disease according to GOLD and LLN5% definitions; ^3^For CCQ, healthy individuals significantly differed from all disease categories (*P* < 0.001); COPD, chronic bronchitis and reversible COPD disorders did not differ significantly (*P* > 0.05); ^4^COPD classification according to GOLD guidelines; ^5^Patients with reversible and irreversible COPD; ^6 ^MRC: Medical Research Council scale for dyspnea.

**Table 4:** Quality of life, obstructive diseases, and smoking types.

Score	Total sample		COPD and Chronic bronchitis subgroup	
Smoking type	Number	Mean (Standard deviation)	Number	Mean (Standard deviation)
Previous smoking				
Never	553	0.45 (0.89)	268	1.33 (1.43)
Cigarette	309	1.56 (1.65)	306	2.15 (1.60)
Waterpipe	55	1.24 (1.41)	33	2.22 (1.40)
Mixed smoking	69	2.21 (1.73)	58	2.95 (1.35)
*P* value ANOVA/Kruskal-Wallis	<0.001*		<0.001^†^	
Current smoking				
Never	553	0.45 (0.89)	268	1.33 (1.43)
Cigarette	479	1.37 (1.52)	513	1.82 (1.49)
Waterpipe	104	0.44 (1.00)	45	1.18 (1.47)
Mixed smoking	80	1.27 (1.55)	51	1.99 (1.57)
*P* value ANOVA/Kruskal-Wallis	<0.001**		<0.001**	

*No significant difference between cigarette and waterpipe; significant difference between any smoking type and mixed smoking (*P* ≤ 0.001); no significant difference between cigarette and waterpipe smokers; **No significant difference between never smokers and waterpipe smokers; no significant difference between cigarette and mixed smokers; ^†^any previous smoker had significantly lower CCQ versus never smokers; mixed smokers had significantly higher values than cigarette and never smokers.

**Table 5:** Quality of life and smoking doses relationship.

Score/smoking type	Number	All sample	*P* value ANOVA	Number	COPD and chronic bronchitis subgroup	*P* value ANOVA	Correlation coefficient
Previous cigarette smoking							
Never smokers	558	0.45 (0.88)	<0.001	267	1.33 (1.44)	<0.001^¶^	0.332^‡^
1–18 pack-years	94	0.99 (1.39)		74	1.65 (1.45)		
18.1–56 pack-years	139	1.42 (1.58)		135	2.06 (1.57)		
>56 pack-years	120	2.71 (1.59)		144	2.84 (1.48)		
Previous waterpipe smoking							
Never smokers	558	0.45 (0.88)	<0.001	270	1.32 (1.43)	<0.001^¶^	0.126^‡^
0.1–29.9 waterpipe-years	42	1.29 (1.54)		26	2.44 (1.36)		
30+ waterpipe-years	67	2.36 (1.69)		59	3.02 (1.28)		
Current cigarette smoking							
Never smokers	617	0.51 (0.93)	<0.001^†^	343	1.30 (1.40)	<0.001*	0.307^‡^
1–18 pack-years	139	0.61 (0.92)		92	1.02 (1.08)		
18.1–45 pack-years	163	1.28 (1.49)		159	1.88 (1.40)		
45+ pack-years	274	2.18 (1.69)		215	2.42 (1.59)		
Current waterpipe smoking							
Never smokers	574	0.45 (0.88)	<0.001^†^	281	1.30 (1.42)	0.001*	0.203^‡^
0.1–20 waterpipe-years	66	0.32 (0.76)		19	0.96 (1.16)		
20+ waterpipe-years	86	1.35 (1.65)		58	2.08 (1.69)		
Current cigarette dependence							
Fagerström 0–5 Low dependence	1259	0.89 (1.35)	<0.001*	833	1.74 (1.56)	<0.001*	0.256^‡^
Fagerström 6-7 Moderate dependence	116	1.39 (1.48)		128	1.93 (1.39)		
Fagerström 8–10 High dependence	108	2.43 (1.65)		149	2.43 (1.56)		
Current waterpipe dependence							
LWDS-11 0–9 Low dependence	74	0.36 (0.66)	<0.001*	33	0.86 (0.87)	<0.001*	0.435^‡^
LWDS-11 10–16 Moderate dependence	40	0.63 (1.06)		21	1.64 (1.39)		
LWDS-11 17+ High dependence	59	1.52 (1.76)		35	2.46 (1.75)		

^†^No significant difference between never and low-level smokers; *no significant difference between low and moderate dependence; ^¶^no significant difference between low and moderate smoking level ^‡^ *P* < 0.001 for correlation coefficients.

**Table 6:** Predictors of lower respiratory quality of life (CCQ).

Factor	Beta	*P* value	Standardized beta	Partial correlation
In all individuals (healthy, COPD and chronic bronchitis)*				
Cumulative cigarette smoking (pack∗years)	0.001	<0.001	0.399	0.404
Older age	0.021	<0.001	0.168	0.155
At least one smoker in the family	0.328	<0.001	0.111	0.129
Lower education	0.126	<0.001	0.108	0.117
Female gender	0.273	<0.001	0.095	0.090
Any heart disease	0.301	<0.001	0.077	0.089
Heating house by diesel	0.205	0.003	0.062	0.083
Cumulative waterpipe smoking (waterpipe∗years)	0.002	<0.001	0.064	0.078
Heating house by hot air	0.281	0.008	0.055	0.066
At least one smoker at work	0.166	0.048	0.044	0.051
Ever lived close to a local power plant	0.11	0.094	0.035	0.048
In all COPD individuals^¶^				
Cumulative cigarette smoking (pack∗years)	0.001	<0.001	0.260	0.263
Inhalation therapy	0.802	<0.001	0.198	0.219
Female gender	0.371	0.002	0.116	0.123
Lower education level	0.165	0.002	0.125	0.123
At least one smoker in the family	0.380	0.003	0.107	0.117
Older age	0.016	0.008	0.115	0.105
Cumulative waterpipe smoking (waterpipe∗years)	0.002	0.003	0.103	0.103
Having a cardiac problem	0.285	0.031	0.08	0.086
Not heating home by central heating	0.315	0.046	0.071	0.080
COPD severity grading	0.097	0.085	0.065	0.069
In nonsmokers^†^				
Lower educational level	0.256	<0.001	0.318	0.272
Any cardiac problem	0.622	<0.001	0.202	0.217
Heating house by hot air	0.433	0.001	0.111	0.123
Older age	0.010	0.011	0.106	0.094
Heating house by diesel	0.201	0.018	0.08	0.089
Ever lived close to a heavy traffic road (<100** **m)	0.215	0.024	0.097	0.084
Occupational exposure to toxic fumes	0.214	0.032	0.072	0.080
At least one smoker in the family	0.106	0.103	0.047	0.059

*R = 0.590 and *R* ^2^ = 0.348 for the model; factors not retained in the model include heating house by butane gas, wood, and central heating, cooking on gas, being occupationally exposed to toxics and ever living close to a heavy traffic road (*P* > 0.05); ^¶^ *R* = 0.500 and *R* ^2^ = 0.250 for the model; factors not included in the model include ever living close to a heavy traffic road, heating house by hot air, by wood, diesel, being occupationally exposed to toxics, ever living close to a power plant, and at least one smoker at work (*P* > 0.05).^†^ *R* = 0.492 and *R* ^2^ = 0.242 for the model; factors not included in the model include gender, ever living close to a power plant, at least one smoker at work, heating its house by butane gas, wood, central heating, and cooking on gas (*P* > 0.05).

## Abbreviations

ANOVA: Analysis of variance
CAT: COPD assessment test
CCQ: Clinical COPD Questionnaire
COPD: Chronic obstructive pulmonary disease
GOLD: Global initiative for obstructive lung disease
MRS: Miscellaneous respiratory symptoms
OR: Odds ratio.
